# Entangled epidemics: tackling vaccine-preventable diseases in the era of frequent epidemics in Africa

**DOI:** 10.11604/pamj.2023.46.32.37485

**Published:** 2023-09-21

**Authors:** Vinie Kouamou, Seth Inzaule

**Affiliations:** 1 Faculty of Medicine and Health Sciences, University of Zimbabwe, Harare, Zimbabwe,; 2 Amsterdam Institute of Global Health and Development, Amsterdam, Netherlands

**Keywords:** Vaccine-preventable disease, emerging outbreaks, immunization

## Abstract

Whilst the largely limited health system and funds are already overstretched while responding to multiple epidemics, ongoing vaccine-preventable diseases (VPD) including polio and measles continue to be a public health threat and expose the weaknesses of the public health system in many African countries. The surge in VPD outbreaks during epidemics appears to be a common trend in Africa, often due to reduced vaccination coverage. The World Health Organization reported that, in 2021, nearly 25 million children missed their first measles dose, 5 million more than in 2019. The drop in childhood immunizations was partly attributed to the COVID-19 pandemic which has caused significant interruption in public health services delivery and reduced vaccination coverage. Vaccines help reduce the incidence of VPD. Therefore, effective VPD outbreak response mechanisms and strategies that include ramping up catch-up campaigns for immunization during epidemic troughs including the provision of vaccines outside clinics as well as assessing newer vaccine delivery models during pandemics are essential to minimize the impact of VPD outbreaks during emerging epidemics. Ensuring access to vaccines to address outbreaks and provide supplemental vaccination is essential if we are to be a VPD-free region.

## Perspective

**Burden of vaccine-preventable diseases during emerging epidemics:** emerging epidemics including Ebolavirus disease (EBV), coronavirus disease of 2019 (COVID-19) and more recently monkeypox disease are major public health threats that continue to exacerbate the vulnerabilities of the African continent [1,2]. The consequences of these emerging epidemics in Africa frequently include socioeconomic instability, disruptions, and breakdown in the healthcare systems including a reduction in mandatory childhood vaccinations [3,4]. Owing to the interruption in the delivery of public health services and reduced vaccination coverage during these epidemics [5], the past 2 years have seen a re-emergence in vaccine-preventable diseases (VPD) including measles and poliomyelitis. Vaccine-preventable diseases (VPD) still continues to be a threat during emerging epidemics, and regrettably continues to exacerbate health inequalities as children are disproportionately affected [6].

**Trend of vaccine-preventable diseases during the 2013-2016 Ebola virus disease outbreak:** the surge in VPD outbreaks during epidemics appears to be a common trend. During the 2013-2016 EBV outbreak, the incidence of measles rose from 0 in 2013 to 108.5 cases per million in 2015 in Liberia, 2.7 cases per million in 2015 to 11.5 cases per million in 2016 in Guinea and 6.9 per million in 2014 to 18 per million in 2015 in Sierra Leone [7] (Table 1). Equally, there were some cases of vaccine-derived poliovirus outbreaks with a general drop in vaccination. These occurrences of VPD during the EBV outbreak highlighted the need to strengthen surveillance systems, enhance vaccination coverage, and ramping-up vaccination campaigns during trough phases of the epidemic. Although efforts have been put in place to design the best public health response in improving prevention and response strategies for future epidemics; regrettably, the re-emergence of VPD continues to be a public health threat in Africa.

**Table 1:** measles incidence by country, during the 2013-2016 Ebola virus disease outbreak

Countries	Year	Confirmed measles	Measles incidence/million population
Liberia	2013	0	0.0
	2014	0	0.0
	2015	436	108.5
	2016	400	97.4
Guinea	2013	39	3.3
	2014	35	2.9
	2015	29	2.7
	2016	128	11.5
Sierra Leonne	2013	13	2.1
	2014	44	6.9
	2015	128	18.0
	2016	195	26.7

**Surge in vaccine-preventable diseases during Ebola virus disease outbreak in the Democratic Republic of Congo in 2018-to date:** fast forward, 2 years after the 2013-2016 Ebola outbreak, a similar rise in children VPD cases was reported in yet another EBV epidemic in the Democratic Republic of Congo (DRC). At the peak of the EBV epidemic in 2019, close to 300,000 measles cases were reported in DRC, with nearly 6,000 deaths [6], a number nearly twice as high as the number of EBV-related deaths reported in the same year (Table 2).

**Table 2:** data on vaccine-preventable diseases during the 2018 Ebola virus disease and 2019 coronavirus disease outbreaks in Africa

Diseases	Characteristics	2017	2018	2019	2020	2021	Up to August 2022
Measles	Total cases	49505	118407	416957	53547	68515	141789
	Cases confirmed	913	26013	11966	4895	5740	24387
	Deaths	528	1200	6562	198	869	1460
	Highest CFR reported	1.2%	1.8%	1.60%	3.30%	1.5%	5.0%
	Countries that reported	4	11	14	14	12	17
Polio	Total cases	0	83	198	378	580	124
	Cases confirmed	0	83	198	378	580	124
	Deaths	0	1	0	0	0	0
	Highest CFR reported	0	12.5%	0%	0%	0%	0%
	Countries that reported	0	4	11	13	16	10
Cholera	Total cases	90640	92631	38673	37248	123943	23768
	Cases confirmed	2160	502	923	165	1754	1514
	Deaths	1969	2090	630	677	3959	386
	Highest CFR reported	6.5%	5.9%	4.90%	4.50%	12.8%	4.10%
	Countries that reported	11	8	6	8	9	9
Yellow fever	Total cases	341	3923	3802	1200	204	5727
	Cases confirmed	32	93	132	87	83	144
	Deaths	45	37	197	35	20	44
	Highest CFR reported	13.2%	26.7%	33.30%	33.30%	20%	33.30%
	Countries that reported	1	5	2	5	7	11

CFR: case fatality rate

**Rise in vaccine-preventable diseases during the COVID-19 epidemic:** reductions in vaccination rates in Africa have played an important role in increasing cases and deaths from measles. The full vaccination coverage rate against measles is still below 50% in Africa, far off from the 90%-95% coverage required to achieve effective herd immunity. Low rates of vaccination coverage in Africa continue to lead to several outbreaks. In 2022, the number of cases of measles has surged to 141,789 with 1460 deaths in 17 African countries as of August 22, majority of the cases and deaths are still from DRC [6] (Table 2).

The surge in measles cases is attributed to a number of factors including reluctance for immunization as well as awareness. The decline in vaccine coverage during COVID-19 has furthermore played a significant role. The World Health Organization (WHO) estimates that up to 25 million children missed their first measles dose in 2020 due to COVID-19 and its mitigation measures [8]. The low vaccine coverage may have resulted in a surge in cases after lockdowns with an increase in population inter-mixing. Besides measles, there has also been a rise in, polio with up to 124 cases being reported in 10 African countries in 2022, 580 in 16 countries in 2021, and 378 cases in 13 countries in 2020 [6]. This reflects a 91-193% increase from pre-COVID levels in 2019 [6] (Table 2).

**Mitigation measures against vaccine-preventable diseases:** Vaccine-preventable diseases (VPD) are a public health crisis with significant economic burden on many African countries, particularly the poorest communities. Their rise since 2020 is greatly concerning and indicates that previous strategies and efforts are still not enough. Identifying and addressing the gaps and challenges that still exist is empirical if we are to be a VPD-free region. It is apparent that VPD outbreaks are bound to occur during epidemics and are a major cause of indirect deaths, especially among children. Learning from past experience, global and national guidance should ensure the continuity of mandatory vaccine programs in every epidemic/pandemic preparedness and contingency plan. The ministry of health of each respective country in Africa supported by WHO and other global partners health stakeholders should re-engage and redouble the efforts to achieve the goals of the Immunization Agenda 2030 [9]. These goals include reducing mortality and morbidity from VPD, ensuring equitable access to vaccines, and strengthening immunization programmes that can withstand significant interruptions during epidemics as well as guidance on maintaining immunization and engaging communities during outbreaks and emergencies. Furthermore, the Immunization Agenda 2030 also contains guidance on implementation and operational research supporting immunization services in the context of emerging challenges.

Another critical aspect is the need to re-mobilize the support from various stakeholders to commit now to avert an even greater crisis in future outbreaks. In line with this, the Africa Center for Disease Control (CDC) is enhancing emergency prevention, preparedness, and response efforts throughout Africa to ensure efficient management of outbreaks [10].

Furthermore, strategies that include ramping up catch-up campaigns for immunization during epidemic troughs as recommended by WHO including provision of vaccines outside clinics, including door-to-door campaigns, at schools, markets, or pharmacies, as well as assessing newer vaccine delivery models during pandemics is critical to minimize the impact of VPD outbreaks during emerging epidemics [11] (Table 3). Vaccines have been effective in almost eliminating VPD globally. However, VPD continues to be a public health threat in many African countries. All children must be vaccinated and effective strategies to increase immunization rate specifically during epidemics are critical if we are to be a VPD-free region.

**Table 3:** proposed solutions, the stakeholders, and the level of implementation

Proposed solutions	Stakeholders	Level of implementation
Global and national guidance on contingency plans to ensure continuity of mandatory vaccine programs during epidemics/pandemics	WHO and partners, public health specialists, Africa CDC, and ministry of health of each respective country	Contingency plans were developed during COVID-19 and adopted in several African countries but there is need for a pathogen-agnostic global and national guidance
Ramping up catch-up campaigns for immunization during epidemic troughs	Ministry of health of each respective country supported by WHO and other global partners	Post-COVID information shows that catch-up campaigns were done but a sustainable mechanism is needed to integrate immunization against vaccine-preventable diseases as part of the epidemic/pandemic response
Assessing newer vaccine delivery models during pandemics	Public health specialists and scientists	Research agenda that still needs to be evaluated
Enhance country adoption of the 2020 World Health Assembly on immunization i.e. Immunization Agenda 2030: a global strategy to leave no one behind which includes guidance for building strong immunization programs that can withstand significant interruptions during epidemics as well as guidance on maintaining immunization and engaging communities during outbreaks and emergencies. The Immunization Agenda 2030 also contains guidance on implementation and operational research supporting immunization services in the context of emerging challenges	Ministry of health with support from WHO and donors	Guidance on national immunization strategies developed and released by WHO in 2021. Progress is ongoing in countries for the adoption of the strategy
Funding is urgently required to commit now to avert an even greater crisis in future outbreaks	WHO, donor and aid community, Africa CDC	Funding ramped up during post-COVID-19 but a sustainable funding mechanism is still required to strengthen immunization services that can withstand significant interruptions during epidemics

WHO: World Health Organization; CDC: Center for Diseases Control; COVID: coronavirus disease
